# Self-Sensing of Piezoelectric Micropumps: Gas Bubble Detection by Artificial Intelligence Methods on Limited Embedded Systems

**DOI:** 10.3390/s25123784

**Published:** 2025-06-17

**Authors:** Kristjan Axelsson, Mohammadhossien Sheikhsarraf, Christoph Kutter, Martin Richter

**Affiliations:** Fraunhofer EMFT, Hansastr. 27d, 80686 Munich, Germany; mohammadhossien.sheikhsarraf@emft.fraunhofer.de (M.S.); martin.richter@emft.fraunhofer.de (M.R.)

**Keywords:** artificial intelligence, drug dosing, embedded system, gas bubble detection, machine learning, piezoelectric micropump, scikit-learn, self-sensing, SMT32Cube.AI, TensorFlow

## Abstract

Gas bubbles are one of the main disturbances encountered when dispensing drugs of microliter volumes using portable miniaturized systems based on piezoelectric diaphragm micropumps. The presence of a gas bubble in the pump chamber leads to the inaccurate administration of the required dose due to its impact on the flowrate. This is particularly important for highly concentrated drugs such as insulin. Different types of sensors are used to detect gas bubbles: inline on the fluidic channels or inside the pump chamber itself. These solutions increase the complexity, size, and cost of the microdosing system. To address these problems, a radically new approach is taken by utilizing the sensing capability of the piezoelectric diaphragm during micropump actuation. This work demonstrates the workflow to build a self-sensing micropump based on artificial intelligence methods on an embedded system. This is completed by the implementation of an electronic circuit that amplifies and samples the loading current of the piezoelectric ceramic with a microcontroller STM32G491RE. Training datasets of 11 micropumps are generated at an automated testbench for gas bubble injections. The training and hyper-parameter optimization of artificial intelligence algorithms from the TensorFlow and scikit-learn libraries are conducted using a grid search approach. The classification accuracy is determined by a cross-training routine, and model deployment on STM32G491RE is conducted utilizing the STM32Cube.AI framework. The finally deployed model on the embedded system has a memory footprint of 15.23 kB, a runtime of 182 µs, and detects gas bubbles with an accuracy of 99.41%.

## 1. Introduction to Piezoelectric Micropumps

**Figure 1:**
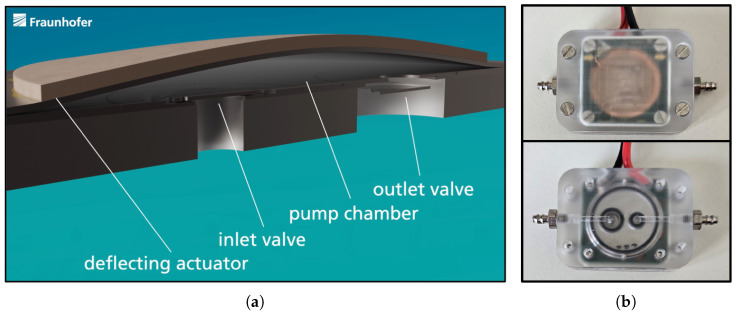
(**a**) Micropump cross-section of pump chamber, inlet and outlet valve, and the piezoelectric deflecting actuator. (**b**) Micropump housing providing electrical contacts and fluidic connections.

Over the past two decades, Fraunhofer EMFT has developed piezoelectric micropumps for the delivery of small volumes of fluids, particularly for drug delivery applications [1,2,3,4,5,6,7,8,9,10]. The steel micropump of 18 mm in diameter consists of a 2 mm thick base plate with inlet and outlet openings (Figure 1a). This base plate holds two stainless steel foils of 25 µm thickness, structured by etching to realize two spring valves for the inlet and outlet. On top of this stack, a 100 µm thin steel foil is placed to enable the realization of a pump chamber later on. This stack is finally bonded through laser welding. As a driving unit, a piezoelectric element composed of lead zirconate titanate, 200 µm thick and 16.2 mm in diameter, is glued on top. To reach a high compression ratio, a patented process, called pretension, is used [11]. With the use of this method, the equilibrium of the piezoelectric diaphragm is in a bent-up position, forming the pump chamber. In order to provide electrical contacts and fluidical connections, the micropump is integrated into a microfluidic housing (Figure 1b). The piezoelectric micropump is driven by a square wave voltage with +300 V for the pump mode (down-stroke) and −80 V for the supply mode (up-stroke). The stroke volume is up to 6 µL depending on the pretension and voltage amplitude. By actuation, the diaphragm moves up and down, which induces over- and under-pressure inside the pump chamber. The pressure peaks cause the check valves to open alternately, creating a volume flow. This results in a flowrate for liquids of up to 9 mL/min in continuous operation at a pump frequency of 25 Hz.

Because gas bubbles can form spontaneously due to the degassing of a fluid, they must always be considered. The Fraunhofer EMFT steel micropumps have a high compression ratio due to the pretension process and are therefore bubble-tolerant [11]. Having a gas-bubble-tolerant micropump opens up the field for reliable drug dosing applications. If the micropump is able to handle gas bubbles, they are not considered as a source of system failure, but they nevertheless affect the dosing accuracy. A standard procedure for drug dosing is the administration of boluses, distributed over several hours or days. If a gas bubble occurs during this protocol, it will result in inaccurate dosing, critical for highly concentrated drugs like insulin. If the dosing system is capable of handling and compensating for the error caused by a gas bubble, the next step is to monitor the amount of gas administered to the patient. Small volumes of air are not relevant, but, if the volume reaches a harmful dose for an adult of 200 to 300 mL, or 3–5 mL/kg, the administration must be stopped immediately [12].

Different approaches have been taken to detect gas bubbles in the micropump system. One approach is to integrate a piezoresistive strain gauge in a Wheatstone bridge configuration before or after the pump chamber, which acts as an inline gas bubble sensor [13,14,15]. However, a gas bubble may be detected by an inline sensor but then be trapped inside the chamber, resulting in an underestimation. Another fault scenario for inline sensors is the overestimation of the gas bubble size if it sticks in front of the sensory element. A more practical solution, especially for drug dosing applications, would be to integrate the sensor inside the pump chamber. This way, it is possible to know exactly whether the volume of a pump stroke is the target drug or a gas bubble. As the integration of a sensor inside the pump chamber is more complex, Zhang et al. [16,17] used the piezoelectric ceramic for the actuation partly as a sensor. The disadvantage of this solution is the decrease in pump efficiency because part of the piezoelectric ceramic is electrically separated and no longer used as an actuator. Other approaches are realized where the piezoelectric ceramic is used as an actuator and sensor at the same time with capacitive strain sensors. In this approach, the piezoelectric ceramic is completely used as an actuator, but the measurement principle is rather indirect [18,19].

In this work, a new approach is presented to detect gas bubbles inside the pump chamber of a piezoelectric micropump without the use of an additional sensor or by modifying, manipulating, or influencing the pump itself. The piezoelectric ceramic is used as an actuator and sensor at the same time. The measurement principle is based on a direct current measurement. This new method is called self-sensing of piezoelectric micropumps. In the following, theoretical background is provided to present the chain of thought regarding the self-sensing principle, although the theory does not play a central role in the final implementation.

Piezoelectric actuators employ the indirect piezoelectric effect to create a mechanical deflection through an applied voltage. In contrast, piezoelectric sensors rely on the direct piezoelectric effect to generate an electrical signal in response to mechanical deformation. The self-sensing micropump utilizes both the indirect piezoelectric effect (actuator) and the direct piezoelectric effect (sensor) simultaneously to achieve the desired self-monitoring capability of the micropump. The indirect piezoelectric effect (1) describes the volumetric displacement V(p,U) in dependency to the pressure *p* and the voltage *U*. The volumetric displacement is proportional to the piezoelectric coupling factor $C_{E}^{*}$ and the fluidic capacity $C_{p}$

(1) $$V\left(p,U\right)=C_{p}\left(p\right)+C_{E}^{*}\left(U\right).$$

The direct piezoelectric effect (2) describes the charges *Q* in dependency of the electrical capacitance $C_{el}$ and the piezoelectric coupling factor

(2) $$Q\left(p,U\right)=C_{el}\left(U\right)+C_{E}^{*}\left(p\right).$$

Both equations are derived from the network theory for the electromechanical coupling of a piezoelectric diaphragm transducer [1,7,8,20,21]. To obtain the current $I_{S}$ (3), we differentiate with respect to time *t*, resulting in a four-term equation

(3) $$I_{S}=C_{el}\frac{dU}{dt}+U\frac{C_{el}}{dt}+p\frac{dC_{E}^{*}}{dt}+C_{E}^{*}\frac{dp}{dt}.$$

The first term represents the current to load regarding the piezoelectric element; the second and third terms represent the large-signal current. The fourth term describes the current induced by pressure changes inside the pump chamber relative to the atmospheric pressure; this is the so-called self-sensing current. A measurement that shows the modulation of the loading current by the self-sensing current is presented in Section 2.

## 2. Concept of a Self-Sensing Micropump

**Figure 2:**
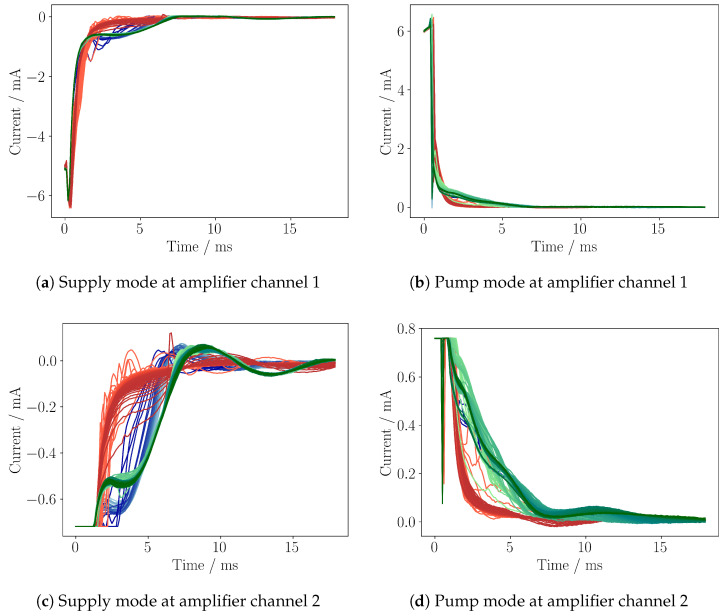
Loading current $I_{S}$ for the supply mode (**a**,**c**) and pump mode (**b**,**d**) of a piezoelectric micropump, driven by a 25 Hz square wave signal (ranging from +300 V to −80 V), with rising and falling edges of a 1 kHz sine wave. Two amplifier outputs with different gains are shown for channel 1 (**a**,**b**) and channel 2 (**c**,**d**). Channel 1 shows the whole signal, while channel 2 has a higher gain to show the relevant self-sensing response. Both channels are sampled by an ADC of STM32G491RE microcontroller with 10 kHz. Each line has a time spacing of 40 ms. Blue-colored cycles indicate the approaching of a gas bubble, red a gas bubble inside the pump chamber, and green the distancing of a gas bubble.

The first proof of concept was realized at Fraunhofer EMFT by Haefner et al. in 2019 and further developed to a first stand-alone prototype by Axelsson et al. in 2022 [22,23]. A measurement of a bubble transition through the microfluidic system with the latest version of the self-sensing electronic is shown in Figure 2a–d.

The pump is driven by a 25 Hz square wave signal with amplitudes of +300 V and −80 V, with rising and falling edges shaped by a 1 kHz sine waveform. A detailed description of the measurement electronics and the testbench setup itself is given in Section 3 and Section 4. The measurement shows the output of both amplifier stages (channel 1 and channel 2) of the amplifier circuit for the supply mode and the pump mode. The gain of channel 1 is configured to show the whole signal, while channel 2 is in saturation, resulting in a cut off at −0.7 mA. Due to the pump frequency of 25 Hz, each subsequent line is 40 ms apart. The measurement is divided into three sections: The first section (blue) shows the approach of a gas bubble. The second section (red) shows the presence of a gas bubble inside the pump chamber. Finally, the third section (green) shows the gas bubble moving away from the pump chamber (Figure 2a–d). Due to the fluid–mechanical couplings of the piezoelectric micropump, the self-sensing current modulates the charging current in a variety of ways so that different system states lead to a characteristic fingerprint in the loading current. The shown measurement demonstrates the strong impact of a gas bubble on the shape of the self-sensing signal. To utilize this effect, an algorithm is required to analyze the self-sensing signal and classify the environmental conditions inside the pump chamber. To realize an implementation on an embedded system to use such an algorithm at product level, an efficient and robust implementation is necessary.

The first and simplest idea is to use a threshold (raw data or parsed) to classify the different signal shapes. However, the study of different pumps with varying driving signals shows that the variance in the data is too broad to define a threshold in a one-fits-all approach (Section 5). Defining the same threshold for these pumps is not possible. Therefore, a more general approach is needed to implement a stable and robust algorithm. Nowadays, artificial intelligence (AI) models are becoming more efficient and can be implemented in limited embedded systems [24,25,26,27]. The ability of AI algorithms to learn complex patterns leads to the idea of training an AI model on different shapes of the self-sensing signal to detect gas bubbles inside the pump chamber.

For an application-oriented use of the self-sensing property, the AI models have to be trained with suitable labeled training data. These data are generated on a testbench especially developed for this purpose and post-processed through cleaning and assignment of labels (Section 4). With these labeled training data, AI models are trained on a NVIDIA A100-SXM4-40 GB graphics card (NVIDIA Corporation, Santa Clara, CA, USA). The training and deployment is realized by a toolchain including TensorFlow, TensorFlow Lite, scikit-learn, ONNX (Open Neural Network Exchange), and STM32Cube.AI. The models are deployed on a microcontroller STM32G491RE (Arm Cortex-M4, 32-bit CPU, 170 MHz, 512 kB Flash, and 112 kB SRAM; STMicroelectronics, Geneva, Switzerland) and evaluated in terms of accuracy, memory consumption, and runtime (Section 5).

## 3. Embedded Self-Sensing Electronics

The embedded system, so-called edge-device, has to perform several tasks in parallel to realize a real-time application of a self-sensing micropump. These tasks are driving a boost-converter circuit to generate high voltage, sampling the amplified loading current of the piezoelectric element, running an AI model to classify the loading current to detect gas bubbles, and finally sending the recorded data and the AI prediction to the PC for storage and live visualization.

For the integration of the self-sensing electronic into a measurement setup, trigger outputs are required to synchronize external sensory data for data post-processing and labeling. In order to realize a flexible and modular development process, debug pins are needed to output the analog and digital signals of the microcontroller.

**Figure 3:**
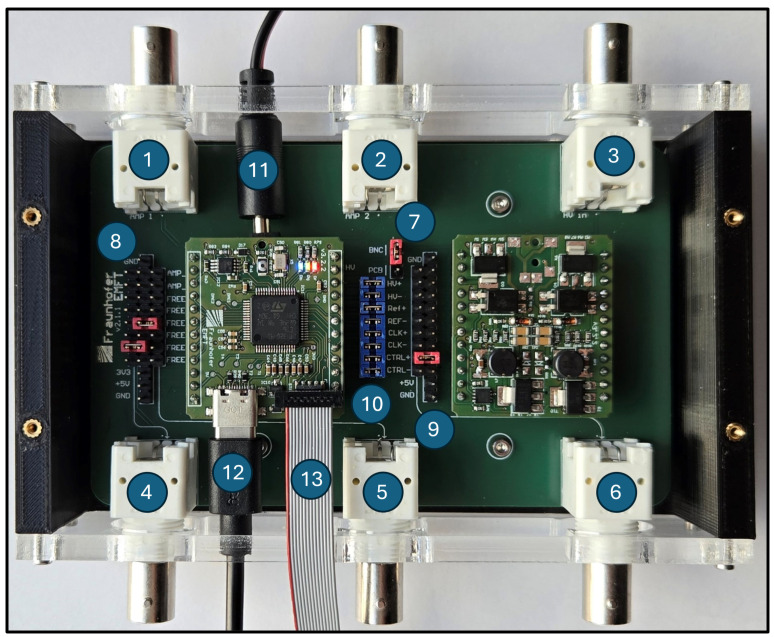
Laboratory adapter with mounted main board (left) and high-voltage generator (right). (1,2) ADC channel 1 and channel 2, (3) external high voltage, (4–6) flexible BNC outputs, (7) jumper to select external or internal high voltage, (8) header rows to configure BNC 4 and 5, (9) pin header row to configure BNC 6, (10) jumpers for signal lines between main board and high-voltage generator, (11) pump connector, (12) USB-C, and (13) STlink programmer.

To address those requirements, the self-sensing electronic (4-layer PCB) is separated into two modules: main board (top layer) and high-voltage generator (bottom layer) (Figure 3). The main board (left) features a microcontroller, self-sensing circuit, pump connector, USB-C port, power supply, and programmer header. The high-voltage generator (right) has a boost-converter, i.e., a DC/DC converter to generate high voltages ranging from +400 V to −100 V from a 5 V input voltage. In order to enable the separation of both modules, the signal lines between top and bottom layers are going over the pin header rows at the side of the PCB. The two modules on top and bottom layers are built independently and then coupled over a laboratory adapter (Figure 3). This adapter enables flexible wiring of the signal lines through jumpers for debugging, as well as signal readout via BNC connectors or directly at the pin headers. The adapter also allows switching from the internal on-board high-voltage generation to an external high-voltage generator.

The foundation of the software implementation is the use of synchronized timers and direct memory access (DMA) to ensure time-correlated measurements and a reduction in the computational cost of the microcontroller CPU. As a clock source, an external quarz with 48 MHz is used. To scale up the system clock to 96 MHz, phase-locked loop of the STM32G491RE is utilized. The embedded high-voltage generation by the boost-converter circuit is driven by two PWM signals at 50 kHz, with a duty cycle of 20%. To independently control the level of the positive and negative high voltages, a feedback loop with two internal comparators and DACs of the SMT32G491RE is implemented for each voltage level. The comparator outputs the PWM signal when the DAC reference voltage is lower than the current high-voltage level. In order to sample the loading current by the ADC with 10 kHz, the ADC peripheral is triggered by an internal timer, which is synchronized with the high-voltage signal. The sampled data are written to an intermediate buffer by DMA. After the data of a complete pump stroke are collected, the AI inference deployed on the STM32G491RE is used to classify the recorded self-sensing signal. The data of the intermediate buffer and the AI predictions are transmitted by USB to a PC.

**Figure 4:**
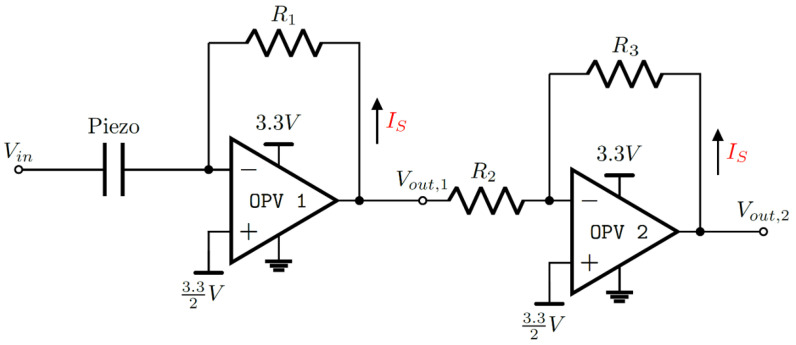
Self-sensing circuit to convert and amplify the loading current $I_{S}$ to a voltage by two operational amplifiers in series. High voltage is applied at $V_{in}$, and the voltage is sampled at $V_{out,1}$ and $V_{out,2}$. The first stage is configured as a current-to-voltage amplifier with a gain of −220; the second stage is configured as voltage-to-voltage amplifier with a gain of −9.96. Both negative inputs are tied to 1.65 V to offset the alternating signal for the microcontroller ADC.

The self-sensing circuit is designed to convert the loading current into a measurable voltage for the microcontroller ADC. Due to the driving of the micropump by the high voltage, the loading current $I_{S}$ with an amplitude of around ±6.5 mA is induced (Figure 2a,b). The features with the highest variance in the self-sensing signal are present in the first 15 ms, with current amplitudes of less than 1 mA (Figure 2c,d). In order to study both the dominant loading current and the much smaller self-sensing response simultaneously, two operational amplifiers (MCP6007) are used. Both amplifiers are used in series with different gain factors to target both current ranges (Figure 4). The resulting voltages are sampled at channel 1 ($V_{out,1}$) and channel 2 ($V_{out,2}$).

The first stage is configured as a current-to-voltage amplifier (4) with a gain of −220, defined by the resistance of $R_{1}$ = 220 Ω. The inverting input of the first operational amplifier is connected to the ground contact of the piezoelectric element.

(4) $$V_{out,1}=-I_{S}R_{1}$$

The second stage is configured as an inverting voltage-to-voltage amplifier (5) with a gain of −9.96, defined by $R_{2}$ = 999 kΩ and $R_{3}$ = 9950 kΩ.

(5) $$V_{out,2}=-\frac{R_{3}}{R_{2}}V_{out,1}$$

To sample both amplifier outputs by the microcontroller ADC, the self-sensing signal must range from 0 V to 3.3 V. To offset both amplifier output voltages, the non-inverting inputs are tied to 1.65 V.

## 4. Testbench Setup and Training Data Generation

**Figure 5:**
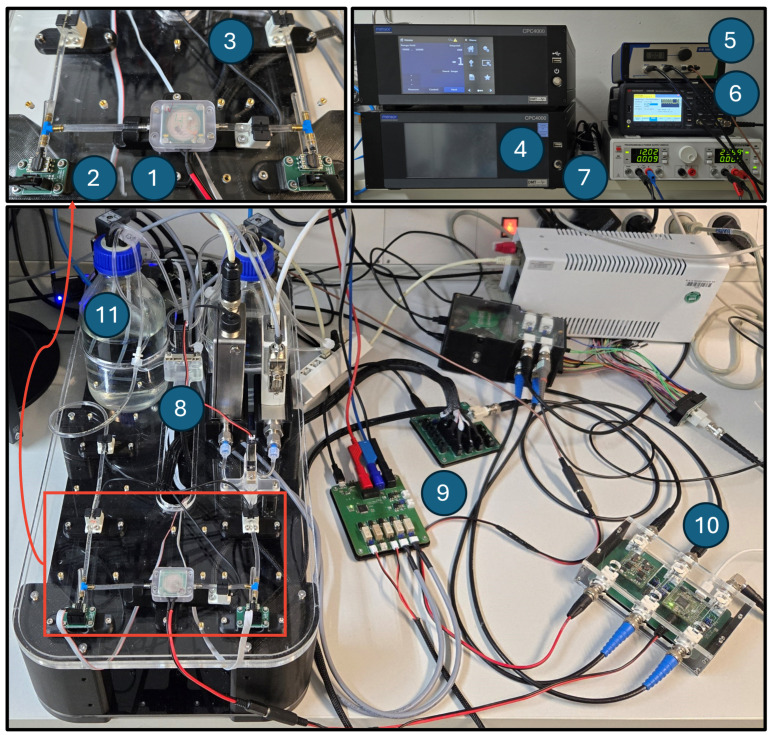
Micropump testbench for simulation of fault scenarios and data generation for AI training. (1) Micropump, (2) pressure sensor Honeywell ABPDRRV015PDSA3, (3) gas bubble detector Panasonic BE-A401P, (4) pressure controller Mensor CPC4000, (5) piezo driver Piezo Jena SVR 1000/3, (6) function generator Keysight K33511B, (7) DC power supply Rohde & Schwarz HM8143, (8) flowmeters Bronkhorst mini CORI-FLOW M14 and EL-FLOW Prestige FG-111B, (9) valve driver and sensor reader, (10) laboratory adapter, and (11) inlet and outlet reservoirs.

The micropump testbench (Figure 5) is designed to generate training data by the simulation of fault scenarios like gas bubble insertion and changes in fore- and backpressure. The testbench is controlled via Python 3.10 interface to enable automated measurement routines. As a central control unit, the edge-device is integrated through a laboratory adapter (10 in Figure 5) to provide trigger signals for synchronization of the high-voltage generation with ADC sampling and sensory data. These simulation operating conditions and their corresponding self-sensing signals are recorded and later used to generate labeled data for training of AI models.

In order to present a repeatable methodology, instead of the embedded boost-converter circuit, an external high-voltage supply is used. The signal is generated by a function generator (K33511B, Keysight Technologies, Santa Rosa, CA, USA) and amplified by a piezo driver (SVR 1000/3, Piezosystem Jena GmbH, Jena, Germany). To drive the piezoelectric actuator safely, a square wave with sine edges for the rising and falling slope is generated utilizing the arbitrary waveform generation of the function generator. For the synchronization, a trigger signal, with the frequency of the target high voltage, is emitted by the main board of self-sensing electronic. Pressure is applied by a single-channel pressure controller (CPC4000, Mensor, San Marcos, TX, USA). To distribute the pressure to the inlet or outlet of the micropump, two 3/2 solenoid valves (Type 0330, Bürkert Fluid Control Systems, Ingelfingen, Germany) are used to connect the inlet and outlet reservoirs either to the pressure controller or to atmosphere. To measure the pressure in the fluidic system, four differential pressure sensors (ABPDRRV015PDSA3, Honeywell International Inc., Charlotte, NC, USA) are used, one for each reservoir and one at the pump inlet and outlet. The pressure measurement is conducted at a sample rate of 10 Hz. Two flowmeters are used to measure the flowrates at the water inlet (Mini CORI-FLOW M14, Bronkhorst High-Tech B.V., Ruurlo, Netherlands) and air inlet (EL-FLOW Prestige FG-111B, Bronkhorst High-Tech B.V., Ruurlo, Netherlands). In order to switch the medium from air to water or to introduce gas bubbles, a 3/2 miniature solenoid valve (Type 6724, Bürkert Fluid Control Systems, Ingelfingen, Germany) is used in the upstream of the micropump. Each inlet of the valve is connected to either air or water at the same pressure level via the inlet reservoir. For gas bubble tracking, four medium sensors (BE-A401P, Panasonic Corporation, Osaka, Japan) are used, one sensor each at the inlet and outlet of the fluidic system and close to the micropump inlet and outlet. PCBs were designed to control the valves by switching 12 V and 24 V from a DC power supply (HM8143, Rohde & Schwarz GmbH & Co. KG, Munich, Germany) and to read out pressure and medium sensors (9 in Figure 5).

For the data generation, one gas bubble at a time is injected into the upstream of the testbench system, and the corresponding self-sensing signal is sampled. These generated datasets are later post-processed and labeled for AI model training. To inject a gas bubble, the continuous operation of the micropump is turned off, the 3/2 valve is switched to air, the pump strokes 20 times, the valve is switched back to water, and the micropump is again continuously actuated. In order to have wide variation in the training data, each pump is driven with different configurations of the high-voltage signal. For the positive amplitude, +300 V, +250 V, and +200 V and for the negative amplitude −80 V, −60 V, −40 V, and −20 V are set. The sine edge of the square wave driving signal is set to 500 Hz and 1 kHz. This parameter space results in 24 different configurations and is repeated for 11 micropumps. For each configuration, three gas bubbles are introduced, one after the other. A single gas bubble transition is visualized in Figure 2c.

**Figure 6:**
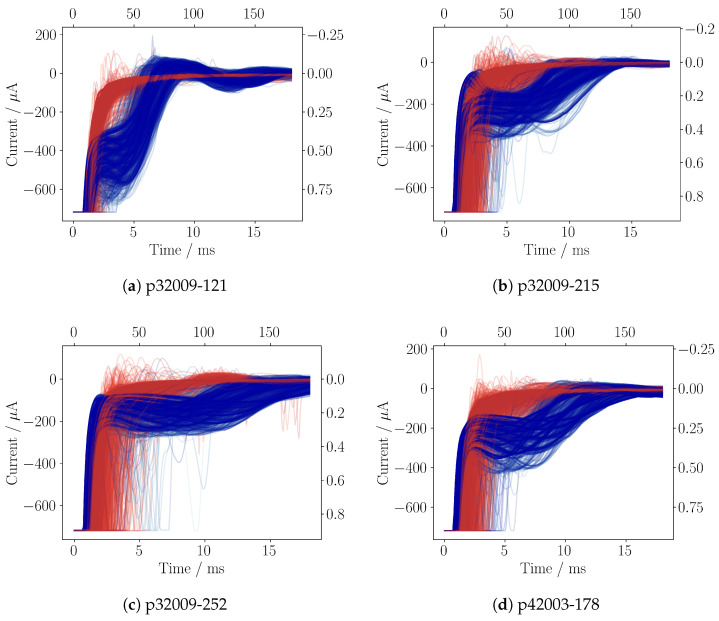
Visualization of four micropump datasets with broad variance (**a**–**d**). Each dataset contains 10k cycles, plotted with light transparency. Blue indicates water inside the pump chamber, while red indicates a gas bubble. The top x-axis shows the number of data points, and the right y-axis shows the normalized input values for AI training.

For the generation of training datasets, the supply mode sampled at channel 2 is used. To label the pump cycles with gas bubbles inside the pump chamber, the medium sensory data are not directly feasible due to the housing, which introduces a dead time between the detection of a gas bubble and its final entry into the pump chamber (Figure 1b). The fact that a gas bubble has an enormous impact on the pressure compensation inside the pump chamber provides the possibility to use an algorithm to separate the pump strokes in terms of their individual distribution of the self-sensing signal. To separate the datasets into two different clusters, a probabilistic Gaussian mixture model (GMM) from the scikit-learn library is used. The algorithm is applied with default configuration on the raw self-sensing data of gas bubble transitions through the pump chamber. GMM assumes that the data points in the two target clusters for water and gas inside the pump chamber are a mixture of a finite number of Gaussian distributions. The parameters of the Gaussian distributions, such as their center and covariance matrices, are unknown. GMM iteratively applies the expectation–maximization (EM) algorithm to fit the distribution parameters to the target clusters [28,29]. Since each gas bubble insertion was recorded separately, the first cycles contain water inside the pump chamber. After the convergence of the EM algorithm, these first cycles are then used to select the water cluster and vice versa the pump cycles with a gas bubble inside the pump chamber. The result of 24 high-voltage signal configurations times three gas bubble insertions yields 72 gas bubble streams, which are concatenated into one dataset for each micropump. The resulting clusters were evaluated visually using t-distributed stochastic neighbor embedding to reduce the high-dimensional data to 2 dimensions [30]. This process was performed for all 11 micropumps; the result of four datasets is shown in Figure 6a–d.

With this method, reliable labeling of the training data is realized. To prepare all 11 datasets for the training routine, each dataset is shuffled and then 5k samples are taken for each label (water/gas bubble) to have evenly distributed datasets. This results in a final set of 11 micropumps: × 2 labels × 5k pump cycles = 110k samples.

## 5. AI Training, Evaluation, and Deployment

For the final application, it is not feasible to train an AI model on every single micropump. An algorithm is necessary that has been trained on a relatively small number of datasets and is robust enough to make accurate predictions on unseen datasets. To evaluate the trained models on unseen micropumps, a cross-training routine is implemented. For one iteration of the routine, 10 out of 11 datasets are used for training. The training dataset (100k cycles) is split into training and test data by 80/20 ratio. In order to calculate the accuracy of the trained model, the remaining dataset is used as the test dataset (10k cycles). This routine is repeated until every pump dataset has been used as a test dataset.

This work investigates machine learning and neural network classifiers, implemented in Python. The machine learning models are implemented from the scikit-learn library. The chosen classifier models are decision trees (DTs), random forests (RFs), and histogram gradient boosting (HGB) classifiers. Neural networks (NNs) and convolutional neural network (CNN) classifiers are implemented from the TensorFlow library. So, five different model classes are trained and evaluated by the above-described cross-training routine. In order to find a suitable configuration for each model, a hyper-parameter grid search is performed.

Scikit-learn models are used in default configuration, while relevant parameters are varied with $P\left(i\right)=\left\{2^{n}\;|\;n=1,2,\ldots ,i\right\}$. For decision tree models, 1050 different configurations are generated by changing the following parameters: max features i=7, max depth i=5, min sample split i=5, and min sample leaf i=6. The random forest configurations go up to 4200 by varying the same parameters as for the decision trees and iterating the number of estimators: 2, 4, 6, or 8. For histogram gradient boosting, only the values for max depth are the same as for DT and RF models. The hyper-parameters of 4000 configurations are as follows: max iter i=8; learning rate: 0.01, 0.05, 0.1, 0.2, and 0.3; max features: 1.0, 0.9, 0.8, and 0.7; and min sample leaf: 20, 40, 60, 80, and 100.

TensorFlow models are trained with learning rate and L2 regularization of 0.001. The epochs are set to 250 with a batch size of 512 and as optimizer Adam algorithm is selected. Early stopping is set to 20, and the option of restoring the best weights is disabled. Activation function of the hidden layers is ReLU, and sigmoid for the output layer. The layers *l* and the number of nodes per layer *n* are implemented with $L_{C}={\left[2^{n}\right]}^{l}$. Layers with descending number of nodes are implemented with $L_{D}={\left[2^{l-i}\cdot n\right]}_{i=0}^{l}$. The configurations for all implemented NN and CNN models are listed in Table 1. The layers of the NN models are implemented as dense layers; this ends up with 47 different configurations. The computational complexity for the CNN is higher due to the implementation through Conv1D layers, so smaller networks are implemented. The CNN kernel sizes of 3, 5, and 7 are evaluated and moved with a stride of 1 and 2; this results in 138 different configurations.

**Figure 7:**
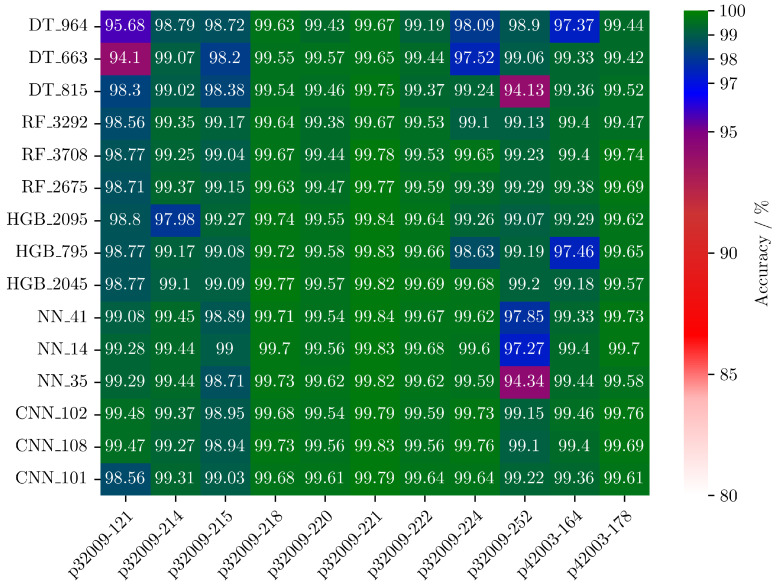
Accuracy of the three best models of each model class: decision tree (DT), random forest (RF), histogram gradient boosting (HGB), neural network (NN), and convolutional neural network (CNN). The accuracy was evaluated due to cross-training with datasets of 11 micropumps.

The training and optimization routine is performed on a NVIDIA GPU A100-SXM4-40GB (NVIDIA Corporation, Santa Clara, CA, USA). After the cross-training is executed, 10 models with the highest accuracy from each of the five model classes are selected. The models are ranked by the worst accuracy of all 11 test datasets. The procedure with these 50 models is repeated and the 5 best models of each class are selected. With these 25 models, the training routine is repeated and the 3 best models of each class are selected. With this final selection, the training routine is finally executed, and the results are shown in Figure 7.

To make a decision on which model to choose for the implementation on the microcontroller STM32G491RE, crucial parameters are accuracy, memory footprint, and computational complexity. In order to characterize all 15 models, they have to be deployed on the embedded system. To convert the Python models into C code, TensorFlow Lite and ONNX are used. TensorFlow models are supported by TensorFlow Lite, and scikit-learn models are supported by ONNX. After the conversion into C code, the models are deployed on a ×86 processor and on STM32G491RE CPU. On the x86 processor, runtime and computational complexity MACC (Multiply And Accumulate operations) are calculated. For the inference on the STM32G491RE, the memory footprint (ROM and RAM) is calculated by STM32Cube.AI framework; the runtime is measured by an internal timer of the microcontroller. In order to validate the correct conversion and deployment, the predictions of the embedded inference are transmitted to the PC and compared with the predictions of the Python inference. The results for all 15 inferences are displayed in Table 2.

The results of the decision tree models have the smallest memory footprint of around 3 kB and the fastest runtime of less than 90 µs. For test datasets p32009-121 and p32009-252, the accuracy drops to 94%, which makes the model unreliable. The memory footprint of the random forest models is 5 to 15, and runtime 2 to 3.5 times higher compared to the DT models. The RF models show the highest and most stable accuracy of the machine learning algorithms. Histogram gradient boosting classifier is close to the random forest, while memory usage ranges from 40 kB to 310 kB. The results of the neural networks are similar to the HGB models with a higher runtime of up to 8 ms. For the evaluation on p32009-252, the accuracy of all three NN models drops to 94%, which indicates overfitting or poor generalization. Convolutional neural networks are as accurate as RF models but are more computationally intensive. The high MACC affects the RAM consumption, which results in runtimes exceeding the defined limits of 40 ms per prediction.

The selected model is the random forest classifier RF 3708 (Table 2). This model is configured with a max depth and max features of 12 and min sample leaf of 16, while the parameters min sample split and number of estimators are set to 8. The average accuracy is 99.41% and ranges from 98.77% to 99.78%, the model has a memory consumption of 15.23 kB and a runtime of 182 µs.

## 6. Discussion and Outlook

This work demonstrates a robust method for optimizing AI models for embedded systems to detect gas bubbles in piezoelectric actuated micropumps. The results show the advantage of machine learning models, in particular random forest, over convolutional neural networks in terms of computational efficiency and memory usage. The drop in accuracy of the neural networks for p32009-252 has to be investigated to eliminate possible overfitting or poor generalization. This could be achieved by varying the batch size and epochs, as well as the learning rate and regularization. For convolutional neural networks, a reduction in memory consumption and a decrease in runtime could be achieved in future developments by implementing quantization-aware training and post-training quantization [25,31,32].

The self-sensing technology presented in this work enables gas bubble compensation for the administration of boluses in miniaturized and portable drug delivery systems that are based on piezoelectric micropumps. By counting pump strokes with a gas bubble inside the pump chamber, these strokes can be compensated for after the gas bubble has left the pump chamber. This is achieved without the need for additional sensors, which increase the cost, size, and complexity of the system.

To utilize the self-sensing technology, a hardware update is required to integrate the operational amplifier circuit. The algorithms are implemented on the same microcontroller that is used to drive the boost-converter for high-voltage generation. As a future perspective, this system could be able to detect not only gas bubbles inside the pump chamber but also faults such as changes in backpressure, breakage of the piezoelectric ceramic, changes in viscosity, or deflected valves. The next steps are to apply the methodology outlined in this work on self-sensing data induced by the embedded high-voltage driver. It should be noted that a pump cycle with the characteristic chaotic self-sensing signal is not synonymous with the case that the entire pump chamber is filled with a gas bubble. It may also be the case that the bubble occupies only a portion of the pump chamber volume and therefore a gas–water mixture is present in the pump chamber. This factor can lead to decreased accuracy, especially with small bubbles. In order to determine the true accuracy of the self-sensing gas bubble detection, a suitable experiment must be designed and conducted.

## Figures and Tables

**Table 1:** NN models with 47 configurations and CNN models with 138 different configurations (kernel size: 3, 5, 7 and stride: 1, 2).

Model	$L_{C}$		$L_{D}$	
	*n*	*l*	*n*	*l*
NN	1,2,…,8	1,2,3,4	8	2,3,…,6
			16	2,3,4,5
			32	2,3,4
CNN	1,2,…,6	1,2,3	8	2,3
			16	2,3
			32	2

**Table 2:** Results of the inference deployment: accuracy (maximum, average, and minimum), memory footprint (ROM and RAM) in kilobytes, computational complexity MACC (Multiply And Accumulate operations), and runtime on a ×86 processor and STM32G491RE CPU in milliseconds.

Model	MCC	Memory [kB]		Runtime [ms]		Accuracy [%]		
		**ROM**	**RAM**	**×86**	**µC**	**max.**	**avrg.**	**min.**
DT 964	372	3.06	0.71	0.003	0.089	99.67	98.63	95.68
DT 663	372	3.14	0.71	0.003	0.089	99.65	98.63	94.10
DT 815	371	2.64	0.71	0.002	0.087	99.75	98.73	94.13
RF 3292	465	46.54	0.71	0.005	0.321	99.67	99.31	98.56
RF 3708	456	15.23	0.71	0.004	0.182	99.78	99.41	98.77
RF 2675	432	15.17	0.71	0.003	0.173	99.77	99.40	98.71
HGB 2095	1000	40.05	0.71	0.007	0.498	99.84	99.28	97.98
HGB 795	5480	310.42	0.71	0.021	3.399	99.83	99.16	97.46
HGB 2045	1000	40.05	0.71	0.004	0.497	99.82	99.40	98.77
NN 41	88,086	342.26	1.70	0.070	7.865	99.84	99.34	97.85
NN 14	16,022	62.01	0.95	0.017	1.477	99.83	99.31	97.27
NN 35	13,846	53.63	0.95	0.013	1.277	99.82	99.02	94.34
CNN 102	514,230	63.01	16.88	0.359	50.241	99.79	99.50	98.95
CNN 108	2,003,286	238.01	33.75	1.401	53.574	99.83	99.48	98.94
CNN 101	2,649,718	102.26	45.00	1.740	56.335	99.79	99.40	98.56

## Data Availability

The raw data supporting the conclusions of this article will be made available by the authors on request.
